# The effects of inpatient suicide on nurses at Weskoppies Hospital: A qualitative study

**DOI:** 10.4102/sajpsychiatry.v30i0.2231

**Published:** 2024-05-21

**Authors:** Nomthandazo Zola, Thandazile G. Mtetwa, Nadira Khamker

**Affiliations:** 1 Department of Psychiatry, Faculty of Health Science, University of Pretoria, Pretoria, South Africa

**Keywords:** inpatient, suicide, nurses, emotional well-being, clinical practice

## Abstract

**Background:**

Inpatient suicide is a serious adverse event in psychiatric wards. Suicide can cause severe trauma to both patients and health professionals, who may develop maladaptation with poor coping skills. Healthcare practitioners are the second victims and historically, this concept has been overlooked. The psychological effects and lack of support have not been sufficiently explored.

**Aim:**

The emotional well-being and clinical practice of nurses who experienced inpatient suicide at Weskoppies Psychiatric Hospital was explored.

**Setting:**

Weskoppies Psychiatric Hospital, South Africa.

**Methods:**

In this qualitative case study, 12 nurses who had lost a patient to inpatient suicide some time during their employment were purposefully selected. Data were collected through individual in-depth interviews, which were audio recorded and transcribed. The data were thematically analysed.

**Results:**

Nurses were negatively affected by inpatient suicide resulting in a range of emotional and psychological effects, including fear, anger, sadness, flashbacks, guilt, and difficulty in coping. Clinical practice factors included being doubtful and extra vigilant. Although nurses received psychological support from the institution, they recommended in-service training with periodic reviews to prevent and manage inpatient suicide.

**Conclusion:**

Inpatient suicide is a serious adverse event, and mental health practitioners become second victims. These events do not only impact the psychological well-being of nurses but also influence the clinical practice. Mental health practitioners should receive adequate training and support in preventing and handling inpatient suicide.

**Contribution:**

This study provided insights into nurse’s perspectives on the effects of inpatient suicide and how they can be supported.

## Introduction

Historically, inpatient suicide rates are higher than that of the general population, with an estimated rate of 147 per 100 000 population,^1^ even though hospitalisation aims to prevent suicide. This alarming statistic highlights the pressing need for effective preventive measures in psychiatric settings. To date, there has not been a consensus on suicide prevention strategies to be used in hospitals because of the complexity of suicide risk assessment.^2^ In the classification of adverse nursing events in a hospital, Chinese inpatient suicide is categorised as accidental and unpredictable.^3^ Suicide fatalities in hospital wards can cause severe trauma to both patients and staff members. Consequently, both groups may develop maladaptation with poor coping skills.^4^

Inpatient suicide is one of the most serious adverse events in both psychiatric and general wards.^1^ However, psychiatric nurses are more prone to witnessing suicide than nurses in other departments.^5^ While suicide has a direct effect on patients or first victims, caregivers and clinicians are also affected and are sometimes referred to as ‘second victims’.^1^ The second victims, such as nurses, may experience varying emotional reactions,^6^ including disbelief, guilt, fear, shame, loneliness, anger, frustration, and job dissatisfaction.^7^ Physically, second victims may experience sleep disturbances, poor concentration, hypertension, and tachycardia among other problems.^8^ The negative emotional reactions following an inpatient suicide may be exacerbated by organisational inquiries and the pressure of attending to police and forensics investigations.

Inpatient suicide deaths have been observed among patients with affective disorders (nearly 50%) and schizophrenia (nearly 30%).^9,10^ In psychiatric hospitals, nurses who work in open wards may be more likely to experience inpatient suicide than those in closed wards, as confirmed in a study conducted in Austria where approximately 80% of suicides occurred in open wards.^9^ This is in contrast with the expectation that serious adverse events are likely to occur in closed wards. Several strategies to prevent inpatient suicide have been proposed. Among others, healthcare personnel should be specifically trained to prevent inpatient suicides,^11^ by learning how to use validated suicide screening and assessment tools.^12^ Psychiatric hospitals should also have appropriate administrative and structural devices in place.^13^ A recent study conducted in the Eastern Cape, revealed a disparity in the knowledge of suicide management, which was linked to a lack of training.^14^ This further highlights the need for authorities to prioritise upskilling of front-line workers in suicide prevention and assessment. Hospital executives should acknowledge the psychological trauma endured by nurses as second victims and provide theory-based educational training, and psychological support programmes to promote nurses’ mental health.^6^

While extensive data and literature about suicide exist in developed countries, this is not the case in developing countries, such as South Africa.^7^ Qualitative studies exploring the psychological experiences of nurses who experience inpatients suicide are even rarer. In 2021, Shao et al.^6^ conducted a meta-synthesis of qualitative studies and included only 11 studies, with only one other study from South Africa. In 2016, Matandela and Matlakala^7^ described the experiences of nurses who experienced inpatient suicide in a general hospital in South Africa.

To the knowledge of the authors, no further studies have explored the experiences of nurses regarding inpatient suicide in psychiatric hospitals in South Africa. This is noteworthy as nurses in psychiatric hospitals are more likely to experience inpatient suicide. In this study, the emotional experiences and clinical practice of nurses who have experienced inpatient suicides in different wards of a specialised tertiary psychiatric hospital in South Africa were explored. The attitudes of these staff members towards suicide prevention and recommendations for suicide prevention strategies were also examined.

## Research methods and design

### Study design

This is a qualitative case study using social constructivism theory where the researcher relied on the views of participants. The case that drew the phenomenon of interest were nurses who had lost patients from inpatient suicide.

### Study setting

This study was conducted at Weskoppies Psychiatric Hospital, in the Tshwane district in Gauteng province, South Africa. This is a tertiary hospital specialising in care and rehabilitation of psychiatric patients of all ages.

### Study population and sampling

Twelve nurses who lost patients because of inpatient suicide during their employment were interviewed. Nurses who had lost patients through suicide and were willing to participate in the study were included. Participants were purposively sampled. Data was collected during individual in-depth interviews.

### Data collection and analysis

The interviewer was the principal investigator, a registrar in psychiatry and this research was part of her master’s degree at the University of Pretoria. Individual in-depth interviews were conducted in English using open-ended questions. The initial question enquired about the events that occurred when the patient committed suicide in the ward. This triggered the recall of the incident, the events that took place, how they coped, and how it affected their well-being and clinical practice. Example of questions included ‘*Could you please tell us about the incident where you lost a patient because of inpatient suicide? How did it affect you as a person?*’

No identifying information was collected; however, basic demographic data were collected (Table 1). Participants were given alphabetic codes (A–L), and the first and last participants were referred to as ‘Participant A (P-A)’ and ‘Participant L (P-L)’, respectively. The interviewer encouraged participants to elaborate when necessary using prompts like ‘Tell me more about that or please elaborate on what you mean by…?’ Field notes were gathered during each interview. All the interviews were recorded and transcribed for further analysis.

**TABLE 1:** Demographic characteristics of 12 nurses who participated in this study.

Demographic characteristics	Value
**Nursing rank**	
Nursing management	3
Professional nurse	7
Auxiliary nurse	0
Assistant nurse	2
**Years of service**	
≤ 10 years	0
> 10 years, < 20 years	4
≥ 20 years	8
**Age in years**	
≤ 30	0
30–40	3
40–50	0
≥ 50	9
**Gender**	
Female	7
Male	5
Other	0
**Current ward**	
Open general	0
Closed general	5
Closed forensic	4
Open forensic	2
Outpatient	1

The transcribed data were thematically analysed and coded. The phases of data analysis included familiarisation with data, coding using different highlighters as codes, searching, reviewing, defining, and naming themes. Common or recurrent themes that indicated data saturation were observed after nine interviews, hence data collection was concluded after 12 recordings. The trustworthiness of the study was established using member checks.

### Intervention

Given the potential of re-traumatisation after participating in this study, a debriefing session and future treatment with a psychologist from another facility was scheduled. Some participants agreed to consult with psychologist, while other mentioned that they had dealt with the trauma.

### Ethical considerations

This study was approved by the University of Pretoria, Faculty of Health Sciences Ethics Committee (ref. 190/2022). Each participant gave written informed consent. Permission to conduct this study was obtained from the National Health Research Database, South Africa, and Weskoppies Psychiatric Hospital management.

## Results

From the interviews with 12 nurses, five inpatient deaths by suicide were identified. Four of these occurred over a decade ago. Some nurses could not recall the exact year; however, they could describe the event in explicit detail, ‘It’s as if it occurred yesterday’. The most recent suicide death occurred in 2020.

Four of these suicide fatalities were because of hanging using different materials and objects from the ward and one was from asphyxia using a plastic bag. Nurses described gruesome details of the incidents.

Five major themes, each with subthemes, were identified from the transcribed data. The major themes included emotional reactions, psychological effects, clinical practice factors, support systems, attitudes about suicide prevention and availability of approved protocols as well as nurse’s recommendations.

### Theme 1: Emotional reactions

Nurses revealed a range of negative emotional reactions. They expressed feelings of sadness, trauma, fear, and anger after the event.

#### Subtheme 1.1: Sadness

The nurses expressed feelings of sadness at the manner of the patient’s death. Most participants commented that patients had poor family support and that they were more like family to them:

‘It was really painful … we felt terrible and heartbroken. It was so heartbreaking.’ (P-B)

‘It was very disturbing … the emotional pain that you feel on that day…’ (P-D)

#### Subtheme 1.2: Trauma

Nurses were traumatised by the site of a hanging body, seeing the cyanosed face of the deceased as well as hearing the screams of the deceased’s family:

‘[*W*]hen it’s a suicide, it becomes more traumatic.’ (P-B)

‘I first didn’t realize that I was traumatized, but the crying, a lot of crying…it was so traumatizing to see that blue face hanging…’ (P-K)

#### Subtheme 1.3: Fear

Nurses reported fear related to the consequences of suicide, and how the management was going to approach the matter. They feared being blamed for negligence and misconduct and ultimately losing their jobs:

‘It was a very scary incident … The first thing that comes to your mind is fear. Fear of being accused that you failed to do your job…’ (P-C)

‘The approach of the management I think is what I was really afraid of. What are they going to say?’ (P-D)

#### Subtheme 1.4: Anger

Some participants expressed feeling angry and becoming irritable following the incident. They were angry because they could not help the patients:

‘I’m still angry, I’m still angry about the situation.’ (P-C)

‘[*S*]ometimes we develop anger, we become irritable at some point.’ (P-A)

### Theme 2: Psychological effects

Aside from emotional reactions, nurses were psychologically affected by inpatient suicide. The psychological effects included sleep disturbances mainly insomnia, flashbacks, as well as difficulties coping with work following the incident.

#### Subtheme 2.1: Sleep disturbances

Participants expressed that they struggled to sleep, and some had to consult their general practitioners for sleeping tablet prescriptions. The sleep disturbance was described as struggling to initiate sleep because of being preoccupied with the suicide event that had transpired in the ward:

‘I couldn’t sleep, I went to my GP … He prescribed medication to sleep.’ (P-H)

‘I realized when I’m sleeping, the flashback of that face that I saw, I even wet my bed because of it.’ (P-K)

#### Subtheme 2.2: Flashbacks

Participants experienced flashbacks of the incident, the face of the deceased, and the screams of the deceased’s family:

‘When you approach that room, the whole memory flashes back like it was yesterday … when you sleep, you feel like you see him hanging like that. You can’t sleep. Sometimes you just wake up and feel like running away because the incident itself is very scary.’ (P-C)

‘Always when I pass the dining hall, I get flashbacks of that patient…’ (P-G)

#### Subtheme 2.3: Maladaptation

Participants struggled to keep working in the same unit where the suicide had occurred. This was exacerbated by the flashbacks of the incident when they were in the ward:

‘I couldn’t cope at all … I couldn’t even cope with going to the ward … and I realized my work was diminishing.’ (P-H)

‘Going back on shift that week was difficult…’ (P-J)

### Theme 3: Clinical practice factors

Themes identified for potential areas of clinical intervention included a sense of guilt, self-blame, and extra vigilance. Participants described a sense of guilt for failing to prevent suicide and they doubted their professional competency. They also expressed that the incident increased their vigilance especially when nursing suicidal patients.

#### Subtheme 3.1: Sense of guilt and self-blame

Participants indicated that they felt as if they had failed the patient. They felt as if they did not do enough to prevent the suicide. Some participants started questioning their professional competency and whether they were in the right career:

‘In a way, you feel you didn’t do enough for the patient.’ (P-A)

‘And it’s like you didn’t look after her enough … you have guilt.’ (P-J)

#### Subtheme 3.2: Extra vigilance

Inpatient suicide had a formative influence on nurses to be more vigilant and review their clinical practice:

‘[*Y*]ou’re actually on the edge … it taught us that there must be frequent checks on patients …’ (P-L)

‘[*I*]t has generated, sort of a concern from my side to be extra careful.’ (P-B)

### Theme 4: Support systems

Some participants had received professional support from the hospital employee wellness psychologist as well as from their managers. Moreover, participants indicated that they supported each other by talking about the incident in their tearoom, which they found to be helpful.

#### Subtheme 4.1: Support from the institution

Participants described varying levels of support from the institution. Some participants had debriefing sessions with in-house psychologists and were supported by their managers:

‘Yes, the hospital offered us counselling sessions with in-house and it helped.’ (P-F)

‘We do have debriefing sessions after the incident, maybe they can also follow up.’ (P-J)

Conversely, some participants indicated that they were never offered such an opportunity. Some nurses felt that one session was not enough, so they hoped to get a follow-up session. For some, these services were not used because of issues of confidentiality or being perceived as weak by their colleagues:

‘The employee wellness services, I don’t think people are keen to attend because of confidentiality.’ (P-A)

‘I got spiritual counselling, but here in the institution they don’t do anything.’ (P-C)

#### Subtheme 4.2: Support from colleagues

Participants acknowledged that discussing the situation with one another helped. They supported each other, which helped to lessen some of the mental distress:

‘Talking amongst ourselves, talking about how we feel helped … we carried each other through that initial phase.’ (P-I)

‘When we are sitting in the tearoom, talking about it helps to heal.’ (P-D)

### Theme 5: Attitudes about suicide prevention and availability of approved protocols

Nurses gave varying reports regarding the availability of approved protocols for suicide at the hospital. Regarding hospital suicide prevention strategies and infrastructure, participants were satisfied; however, they denoted that some units still had exposed plumbing pipes that might potentially aid hanging. They also reported that the CCTV cameras in some units had blind spots, which might compromise monitoring.

#### Subtheme 5.1: Uncertainty about the availability of protocols

Participants were uncertain about the availability of written approved protocols for suicide prevention at the institution:

‘Honestly we don’t have a formal thing.’ (P-H)

‘For suicide, I shall think it might be in the ward, in the file somewhere.’ (P-D)

#### Subtheme 5.2: Nurse’s attitudes about suicide prevention

Nurses felt that suicide prevention strategies should be employed by the institution. They commented that they found CCTV cameras to be very helpful. Nurses also mentioned CCTV camera blind spots that could potentially be used by patients to commit suicide:

‘Structurally, I cannot say we are 100% but they have tried.’ (P-G)

‘For now we are doing well … and with cameras … you can constantly monitor what they are doing.’ (P-J)

### Recommendations

Nurses recommended additional suicide prevention measures and support. They felt that there was a need for continual training in suicide prevention and a need for treatment of suicidal patients. Nurses also mentioned that there was need for adequate staffing on night and weekend duty because most suicides occurred during these periods when there were limited staff on duty. They recommended that security officers be appointed to consistently monitor CCTV as this would enable them to perform other duties efficiently. Nurses suggested a need for follow-up consultations with psychologists after initial debriefing sessions. Finally, they requested the hospital management to review the infrastructure to minimise any potential hazards.

## Discussion

This study describes the effects of suicide on the emotional well-being and clinical practice of psychiatric nurses at a large psychiatric hospital. Nurses recounted their experiences of inpatient suicides, even though some had occurred more than a decade ago. Nonetheless, they recalled a range of negative emotions and psychological sequelae.

In this study, nurse’s mental health was adversely affected by inpatient suicide. The negative effects included anger, fear, sadness, flashbacks of the incident, and sleep disturbances, including nocturnal enuresis. These findings are consistent with previous research. Gulfi et al. and Matandela et al. observed that the suicide of a patient can affect the mental health of practitioners in both personal and professional aspects.^7,15^ Flashbacks and nocturnal enuresis were novel findings in our study. This adds emphasis to the magnitude of the mental health effects of inpatient suicide on health practitioners. Tan et al. added emphasis on health practitioners being second victims of inpatient suicide.^3^

While the clinical practice of nurses was negatively affected by inpatient suicide, it also had a formative influence. This study confirms Matandela et al. who also found that inpatient suicide resulted in a diminished sense of professional competency, including feelings of guilt.^7^ Gulfi et al. also observed that mental health practitioners who experienced inpatient suicide doubted both their skill and their ability to prevent suicidal behaviour.^15^ Beyond the adverse effects, inpatient suicide had a positive influence on the nurses in this study, motivating them to be vigilant and frequently review patients. This is in line with the existing research that found that inpatient suicide may motivate professionals to review and improve their professional practice.^15^

Although nurses were supported through debriefing sessions by the institution’s employee wellness psychologist and their managers, some participants did not utilise these services for fear of being perceived as weak or possible breach of confidentiality. This highlights the issue of stigma in mental health among health professionals, which indicates an interesting area of future research. Studies show that despite mental health promotion and advocacy, the stigma persists and plays a huge role in limiting health-seeking behaviour for desperately needed mental health treatment.^16^ The psychiatric field is facing a growing interest in associative stigma, whereby they are often viewed with the same stigmatising preconceptions as their patients.^17^ This may further aggravate stigma in mental health.

Even though participants reported uncertainty about the availability of approved protocols for suicide prevention in the institution, they indicated that having CCTV cameras improved suicide prevention as this facilitated closer monitoring of patients. While CCTV cameras in rooms may infringe on a patient’s right to privacy, clinical practice must balance this right with the ethical code of beneficence.^18^

In light of this study, areas of future research may include an assessment of the confidence of mental health care practitioners in treating a suicidal patient, as well as informed ways to eliminate the stigma among mental health professionals and across the medical field, as this is still a prominent issue.

This study concludes with recommendations for: (1) Having written approved protocols for suicide prevention readily available for nursing personnel, (2) adequate training of nursing staff with regular updates on dealing with inpatient suicide, and (3) assurance of sufficient psychological support for nurses by the employee wellness team. The magnitude of the effects of inpatient suicide on nurses should not be underestimated. These recommendations would help minimise the emotional trauma experienced by the nurses after losing a patient from inpatient suicide. The limitations of this study were the small sample size and that it was conducted in one institution.

## Conclusion

Inpatient suicide is a serious adverse event that leaves both patients and medical staff with emotional trauma. Nurses reported a range of negative emotional reactions, psychological effects, and professional effects. It also had a formative effect on nurses, motivating them to be vigilant. The issue of stigma among mental health professionals affecting their help-seeking behaviour was noticed. This highlights the need to continue with means to shatter mental health stigma especially among mental health practitioners. It is therefore vital to have adequate support for nurses, including clear and precise formalised protocols and in-service training on how to deal with suicide emergencies.
